# Omega-3 Fatty Acids in Early Prevention of Inflammatory Neurodegenerative Disease: A Focus on Alzheimer's Disease

**DOI:** 10.1155/2015/172801

**Published:** 2015-08-02

**Authors:** J. Thomas, C. J. Thomas, J. Radcliffe, C. Itsiopoulos

**Affiliations:** ^1^Department of Physiology, Anatomy and Microbiology, La Trobe University, Bundoora 3086, VIC, Australia; ^2^Department of Rehabilitation, Nutrition and Sport, La Trobe University, Bundoora 3086, VIC, Australia

## Abstract

Alzheimer's disease (AD) is the leading cause of dementia and the most common neurodegenerative disease in the elderly. Furthermore, AD has provided the most positive indication to support the fact that inflammation contributes to neurodegenerative disease. The exact etiology of AD is unknown, but environmental and genetic factors are thought to contribute, such as advancing age, family history, presence of chronic diseases such as cardiovascular disease (CVD) and diabetes, and poor diet and lifestyle. It is hypothesised that early prevention or management of inflammation could delay the onset or reduce the symptoms of AD. Normal physiological changes to the brain with ageing include depletion of long chain omega-3 fatty acids and brains of AD patients have lower docosahexaenoic acid (DHA) levels. DHA supplementation can reduce markers of inflammation. This review specifically focusses on the evidence in humans from epidemiological, dietary intervention, and supplementation studies, which supports the role of long chain omega-3 fatty acids in the prevention or delay of cognitive decline in AD in its early stages. Longer term trials with long chain omega-3 supplementation in early stage AD are warranted. We also highlight the importance of overall quality and composition of the diet to protect against AD and dementia.

## 1. Introduction

Alzheimer's disease (AD) was first described in 1906 by German psychiatrist Alois Alzheimer, who observed abnormal clumps and tangled bundles of protein in the brain of a patient who experienced memory loss, language difficulties, and abnormal behaviour [1]. The risk of developing AD increases exponentially with age and is the leading cause of dementia and the most common neurodegenerative disease in the elderly; prevalence rates in 65–74 year olds are estimated to be 3%, rising to 19% for 75–85 year olds, and nearly 50% in those aged over 85 [2]. AD is more common among older people but it is not a normal part of ageing. As the global population ages, the prevalence of AD is expected to rise from 36 million to 115 million sufferers by 2050 [2].

Dementia imposes a huge economic burden, both through direct (medical and social care) and indirect (unpaid caregiving by friends and family) costs. In 2010, the estimated worldwide cost of dementia to society was US$ 604 billion, of which 89% was incurred in high income countries [2]. A UK study commissioned by the Alzheimer's Research Trust identified that the societal costs of dementia (£23 billion) were almost equivalent to those of cancer, heart disease, and stroke combined; yet dementia research attracted only 2% of the funding that cancer and heart disease research attracted [2]. Given that the dementia epidemic will continue to rise worldwide alongside diabetes and cardiovascular disease, the economic burden of care will become untenable. Therefore, a focus on prevention or early intervention is urgently needed. Although the recent National Institutes of Health State-of-the-Science conference on Alzheimer's prevention reported that “firm conclusions cannot be drawn about the association of any modifiable risk factor with cognitive decline or AD,” there was a clear message that the evidence was insufficient, trials were focused on older participants with late stage disease, and further research was urgently needed in early onset disease where the impact is likely to be greater [1]. This review specifically focusses on the evidence supporting the role of long chain omega-3 fatty acids in the prevention or delay of progression of AD in its early stages.

## 2. Currently Known Risk Factors for Alzheimer's Disease

The nonmodifiable risk factors for AD are well established and include advancing age, genetic factors (such as the presence of the APOEe4 allele), and family history [24]. To date, there is no high level evidence to support or confirm that any modifiable risk factor (such as nutritional supplements, herbal preparations, diet, prescription and nonprescription medications, social or economic factors, medical conditions, and toxins or environmental exposures) is associated with reduced risk of AD [1]. Although multiple studies have shown that diet and lifestyle factors are associated with risk for AD, the scientific evidence is lacking and further studies are needed. Chronic diseases such as obesity, diabetes, hypertension, hyperlipidaemia, and depression have been associated with AD [24].

Due to an increasing body of evidence suggesting an association between diabetes and increased risk of neurodegenerative diseases such as AD (with AD itself being suggested as type 3 diabetes), it is essential to explore potential therapeutic interventions. Although the underlying pathophysiology and mechanistic aspect of diabetes-induced cognitive impairment still remain poorly understood, therapeutic interventions to delay or prevent the onset of cognitive impairment due to disease conditions, such as diabetes, need to be explored. It is evident from the existing literature that onset of cognitive impairment in diabetes and neurodegenerative conditions such as AD could have major underlying processes that cause neuronal death: these include defective antioxidant defence mechanisms, inflammatory processes, and reduced mitochondrial energy production. A nonpharmacological treatment approach is preferred because of the nature of its safety and efficacy. Trials that have used long chain polyunsaturated fatty acids (PUFAs) demonstrate an array of beneficial effects, particularly improving cognitive function in patients with early onset mild cognitive impairment [15, 16, 22].

At the recent International Conference on Nutrition and the Brain (Washington DC, July 2013), an expert panel developed dietary and lifestyle guidelines for the prevention of AD based on the best available evidence [24]. The panel agreed on seven guidelines which include minimising intake of saturated fats and trans fats; increasing intake of vegetables, legumes, and fruits; sourcing Vitamin E from foods not supplements; increasing intake of B12 through fortified foods and supplements; choosing multivitamins without iron; avoidance of cookware or other products containing aluminium; and including aerobic exercise of 40 min three times per week.

Interestingly, this consensus report by Barnard and colleagues [24] on diet and lifestyle guidelines did not address the importance of omega-3 fatty acids, or intake of fish, on AD risk. Multiple consensus documents and recent reviews [1, 2, 25] have identified that the most consistent evidence on nutrition and AD risk is for longer chain omega-3 fatty acids, primarily obtained through regular fish consumption; however recent supplementation trials are promising. Long chain omega-3 fatty acids, including eicosapentaenoic acid (EPA; 20:5n-3) and Docosahexaenoic acid (DHA; 22:6n-3), are predominantly sourced from marine fish [26]. EPA and DHA can also be synthesised from *α*-Linolenic acid (ALA; 18:3n-3), which is present in a number of green leafy plants, seeds, nuts, herbs, and oils, such as flaxseeds, walnuts, soybean oil, canola oil, and hempseed oil [27]. A low conversion efficiency of ALA into EPA and DHA, which varies between individuals, has been reported in a number of studies (e.g., see review [28]). The conversion efficiency of ALA to EPA varies between 0.2% and 21%, and that of ALA to DHA varies between 0% and 9% [29, 30]. This suggests a minor role for ALA in reducing AD risk and, therefore, the emphasis of our review is on EPA and DHA omega-3 fatty acids.

## 3. What Is Alzheimer's Disease?

### 3.1. Pathophysiology of Neurodegenerative Diseases

Neurodegenerative diseases represent major unmet challenges for therapeutic intervention. Neurodegenerative disorders can be considered in 3 main groups: (1) protein misfolding disorders, (2) mechanical injury and ischemia-reperfusion injury, and (3) myelin and lipid storage disorders [31]. These disorders arise from inflammatory, neurodegenerative, metabolic, or ischemic primary insults [32]. Generally, the risk of developing a neurodegenerative disease increases with aging. They result from failure in brain connectivity, which is formed by neuronal-neuronal, neuronal-glial, and glial-glial contacts [33]. This review focuses on Alzheimer's disease, a protein pathology disease.

### 3.2. Genetics, Clinical Trajectory, and Major Histopathology of Alzheimer's Disease

The exact etiology of AD is unknown, but environmental and genetic factors are thought to contribute. Genetic factors play a significant role in familial AD. This rare autosomal, dominant disease with early onset (<65 years) is caused by mutations in the genes encoding amyloid precursor protein (APP) and presenilin (PSEN1), both linked to amyloid-*β* metabolism [34]. Familial AD accounts for about 1–5% of all AD cases [35]. For late onset AD (>65 years; representing 95–99% of all AD cases), the relevance of specific genetic mutations is not clear. For this sporadic form of AD, it is generally accepted that epigenetic components (ageing, genetic, and environmental risk factors) play a more dominant role [35].

**Figure 1:**
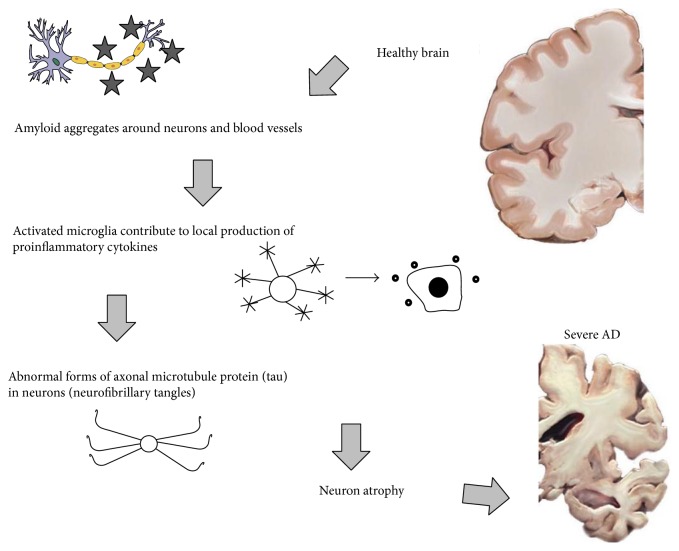
Schematic representation showing key pathological features of the degenerative process in Alzheimer's disease (AD). The common characteristics are (1) amyloid-*β* plaques and (2) neurofibrillary tangles. Neuroinflammatory changes (3) have been identified as the third important component of the disease. Microglia migrate to the plaques and enhance amyloid-*β* deposition with chronic activation.* Brain and neuron images from Wikimedia Commons (* http://upload.wikimedia.org/wikipedia/commons/c/cc/Alzheimers_brain.jpg. http://upload.wikimedia.org/wikipedia/commons/b/be/Derived_Neuron_schema_with_no_labels.svg *).*

AD is characterized by cognitive alterations, memory loss, and behavioural changes which affect daily living.  Table 1 records the well-characterised stages of slow but progressive AD development. The predominant theory for the degenerative process in AD (summarised in Figure 1) subscribes that the deposition in the brain of highly insoluble amyloid-*β* occurs early in AD and suppresses synaptic plasticity. Disruption of dendritic spine formation thereby interferes with memory consolidation. The formation of amyloid plaques also activates glial cells to augment inflammation in the brain. Subsequent signalling events trigger abnormal intraneural formation of hyperphosphorylated tau protein (pTau), resulting in neurofibrillary tangles years/decades later [36]. At autopsy, AD postmortem brains share a number of common features. These include significant cortical atrophy and secondary ventricular enlargement. Neuropathogenic assessment reveals the highly insoluble neuritic plaques (amyloid-*β*; extraneuronal) and neurofibrillary tangles (tau; intraneuronal). AD brains are also characterized by prominent reactive astrogliosis due to destruction of nearby neurons [37].

## 4. Role of Inflammation in Neurodegenerative Diseases

### 4.1. Acute versus Chronic Inflammation in the Brain

The brain is populated by 4 types of cells: microglia, astrocytes, neuron, and endothelial cells. Behind the blood brain barrier, there is an absence of leukocytes and antibodies which constitute key defence cells in peripheral immune system. Instead, microglia are the brain's resident immune cells capable of phagocytosis. They mount localized inflammatory response and have both apoptotic and neurotoxic actions. Initial triggers for neurological diseases may differ considerably, but the subsequent pathways that involve inflammatory processes and involve brain damage often share crucial pathological mechanisms [38]. In an acute insult/stress scenario, pathogens, protein aggregates or damaged neurons may activate inflammatory microglia and, appropriately, they take on the morphology of activated macrophages to remove the threat [39]. In contrast, sustained low grade brain inflammation (chronic microglial activation) may contribute to the ongoing pathology of neurodegenerative disease (e.g., Alzheimer's disease, Parkinson's disease, Multiple sclerosis). Various proinflammatory mediators are increased in the brains of patients with neurological disorders and have been shown experimentally in animal models. These inflammatory factors are thought to contribute to the damage and subsequent loss of neurons [32, 40]. Since prolonged low grade inflammation can lead to loss of neurons in the brain, one hypothesis is that early prevention or management of inflammation could delay the onset or reduce the symptoms of AD.

### 4.2. Inflammation in Alzheimer's Disease

Among the protein misfolding disorders, AD has provided the most positive indication to support that inflammation contributes to this neurodegenerative disease. In AD, there is no obvious accumulation of activated immune cells in the central nervous system. However, potent inflammatory molecules such as cytokines/cytokine receptors are detected in cerebral spinal fluid and plaques from AD patients [37, 38]. The amyloid-*β* plaques have been demonstrated to increase proinflammatory cytokines and reactive oxygen species, in addition to neurotoxic secretory products [37]. Changes in microglia morphology have been described (from ramified (resting) to amoeboid (active)) and astrogliosis has been observed surrounding senile plaques [41]. Moreover, long-term use of anti-inflammatory drugs (NSAIDS) reduces the risk of developing AD [42] (decreased plaque burden pathology/increased cognitive function/linked to reduced microglial activation), supporting therapeutic immune-modulatory approaches against AD progression. An important note is that these benefits of NSAID treatment cannot be obtained in established AD [43]. This implies that inflammation precedes neuronal loss in the disease. We have previously shown that DHA supplementation reduces markers of inflammation in the hippocampus [44].

## 5. Is Alzheimer's Disease Type 3 Diabetes? 

For decades, AD has been commonly attributed to amyloid-*β* and pTau aggregation. An emerging body of evidence, led by neuropathologist de la Monte [45], suggests that people that have insulin resistance, in particular those with type 2 diabetes, have an increased risk of suffering from AD, estimated between 50% and 65% higher. Whether primary or secondary in origin, brain insulin/insulin-growth-factor resistance initiates a cascade of neurodegeneration that is propagated by metabolic dysfunction, increased oxidative and endoplasmic reticulum (ER) stress, neuroinflammation, impaired cell survival, and dysregulated lipid metabolism. Lack of insulin not only impairs cognition but seems to be implicated in the formation of the amyloid plaques [45]. Additionally, insulin resistance with subtle cognitive impairments is seen even in normal adults with prediabetes, suggesting a higher risk of developing AD in later life [46]. Insulin resistance in the brain has been shown to compromise survival of neurons, metabolism, and neuronal plasticity which are critical for memory formation and normal cognitive function [47].

The largest epidemiological studies conducted so far, the Rotterdam [48] and Hisayama [49] studies, indicate that patients suffering from diabetes have increased likelihood of developing cognitive impairment as seen in dementia. The relationship between diabetes and dementia has been clearly established by Velayudhan and coworkers [50], where diabetic patients progressed to dementia after a 4-year followup period. These results indicate a clear need for early screening of mild cognitive impairment (MCI) in diabetes patients, as MCI has been suggested to be the earliest detectable stage of AD [51]. An early screening for MCI in diabetic patients would also be beneficial in modifying lifestyle-related factors and creating the potential for dietary intervention or supplementation such as with omega-3 fatty acids to ameliorate AD disease progression.

## 6. Existing Pharmacological and Herbal Treatments for Alzheimer's Disease

There are currently over 1400 registered clinical trials investigating the effect of drugs or single nutrient supplements on the development/progression of AD [52].

In early stage AD, current approved drug therapies include the cholinesterase inhibitors (e.g., Donepezil (Aricept)). In moderate to advanced stage AD N-methyl D-aspartate receptor antagonists (e.g., Memantine (Ebixa)) are commonly used. Although these drugs improve symptoms in the short term they invariably do not inhibit progression of AD and are often associated with multiple side effects including nausea, diarrhoea, insomnia, tremor, muscle cramps, and occasionally hallucinations [53].

Over the last decade there have been an excess of 28 systematic reviews published on the Cochrane database investigating the evidence for the beneficial effects of supplementation with B-group vitamins (folate, B-12, thiamine, B-6), Vitamin E, or herbal medicines such as ginkgo biloba and ginseng on cognitive decline in AD. The evidence for the potential benefit of these nutraceuticals on dementia in AD has been systematically reviewed and evaluated against the National Health and Medical Research Council (NHMRC) of Australia levels of evidence by Kotsirilos and colleagues [54]. The authors conclude that there is Level 1 evidence (equivalent to meta-analyses and systematic reviews) for the benefits of fish oil and the Chinese herb ginko biloba; Level 2 evidence (equivalent to findings from randomized controlled trials (RCTs)) for the Ayurvedic herb brahmi (Bacopa Monnieri), sage, lemon balm, turmeric, ginseng, CoQ10 (Coenzyme Q10), Vitamin D, and magnesium. These nutraceutical therapies require careful evaluation of the patient's health status and medication as drug-nutrient and drug-herb interactions can occur [54].

Patients with AD are often malnourished and present with multiple nutrient deficiencies. Therefore, correcting nutrient deficiencies, particularly the B-group vitamins and Vitamin D, and improving nutritional status are clearly important in prevention of AD progression. As noted previously, normal physiological changes to the brain with ageing include depletion of major long chain omega-3 fatty acids, which constitute 30–35% of the brain, and this process is accelerated in neurodegenerative conditions such as AD [55]. The long chain omega-3 fatty acids are concentrated in the phospholipid membrane of the brain, particularly at the synapses [56], and brains of AD patients have lower DHA. Therefore, there appears to be a strong physiological rationale to supplement patients with AD with the long chain omega-3 fatty acid DHA.

## 7. Epidemiological Evidence for the Role of Dietary Intake and Alzheimer's Disease

### 7.1. Evidence from Observational Studies

Cross-sectional studies provide evidence of a relationship between diet quality and prevalence of AD [3–8]. Lower intake of dietary nutrients was reported using food records and/or 24-hour diet recalls in “early stage” AD patients (*n* = 36) compared to age-matched cognitively intact subjects (*n* = 58) [3]. Nutrient intakes identified as being significantly lower in patients with AD included energy, all macronutrients, calcium, iron, zinc, Vitamin K, Vitamin A, dietary fibre, omega-3 fatty acids, and omega-6 fatty acids [3]. Dietary patterns were also assessed in 2,148 elderly subjects, of which 253 developed AD. A dietary pattern which exhibited a lower risk on development of AD was identified and was comprised of higher intakes of salad dressing (not specified, but presumably olive oil and/or vinegar-based), nuts, fish, tomatoes, poultry, cruciferous vegetables, fruits, and dark and green leafy vegetables; the diet also contained a lower intake of high-fat dairy products, red meat, organ meats, and butter [4].

Some epidemiologic studies suggest that higher dietary intake of antioxidants, vitamins B6, B12, and folate, unsaturated fatty acids, and fish are related to a lower risk of AD, but reports are inconsistent [57]. Modest to moderate alcohol intake, particularly wine, may be related to a lower risk of AD [7]. The Mediterranean diet may also be related to lower AD risk [8]. The traditional Mediterranean diet is characterised by an abundance of bioactive phytonutrients, with antioxidant and anti-inflammatory potential, derived from extra virgin olive oil as the main added fat, fresh fruits and leafy vegetables, legumes, wholegrain cereals, nuts and seeds, fish and red wine, with moderate portions of meat and dairy [58]. This diet provides a model that delivers a rich anti-inflammatory diet in a palatable cuisine.

A recent systematic review of 11 prospective studies worldwide examined the link between a Mediterranean-type diet and cognitive decline (including AD) [9]. Amongst 4 prospective studies that focussed on AD incidence, the review reported a 28–48% reduced risk of developing AD in response to higher level of adherence to a Mediterranean-type diet. Furthermore, higher adherence to a Mediterranean-type diet was associated with a 73% lower risk of dying of the disease in participants who already had clinically diagnosed AD [9]. A recent meta-analysis on 133,626 people within 3 prospective cohort studies also showed that closer adherence to a Mediterranean diet led to a 13% lower incidence of neurodegenerative diseases such as Parkinson's disease and AD [10].

Whilst dietary patterns are of interest, this review is focused on dietary fats in relation to prevention of AD. Dietary fat alone has been reported to have a role in the prevention of AD onset. Laitinen and colleagues [5] found that polyunsaturated fats were associated with decreased rates of dementia and AD. This data was derived from a 21-year followup of 1,449 individual original study participants. Saturated fats were associated with an increase in rates of dementia and AD [5]. In relation to the role of polyunsaturated fats in particular, epidemiological evidence exists showing a role for omega-3 fatty acids in reducing the onset of AD. A case-cohort study showed a 72% reduction in the odds of developing AD in those with the highest tertile of dietary DHA. Fish intake was also associated with lower odds of developing AD, but this did not reach statistical significance [6].

### 7.2. Evidence from Dietary Interventions

Whilst there are a number of observational studies assessing the association between diet and the risk of neurodegenerative diseases such as AD [3–6, 57], there are no published dietary intervention RCTs investigating the effect of diet, including omega-3 rich food such as oily fish, and the onset of AD. Dietary intervention studies have instead assessed markers of AD.

Dietary intervention RCTs previously conducted in relation to the development of AD (see Table 2), include a study comparing a diet high in saturated fat and high glycemic index (GI) carbohydrates to a diet low in saturated fat and GI [11]. Bayer-Carter and colleagues [11] measured cerebrospinal fluid markers of AD and tested cognition in 20 healthy adults and 29 adults with amnestic mild cognitive impairment (aMCI). Participants receiving a diet low in saturated fat and low GI for 4 weeks exhibited a decrease in markers associated with disease risk of AD, including central nervous system levels of amyloid-*β* 42 (A*β*-42), compared to the high fat and high GI intervention. Lipoproteins, oxidative stress, and insulin levels were also decreased in response to the low saturated fat and low GI diet [11]. A similar finding in relation to acute effects of dietary fat was reported by Karczewska-Kupczewska and colleagues [12]. These researchers showed that a single high fat meal (Calogen), in which energy comes almost in total from fat (450 kcal/100 mL), decreased circulating brain-derived neurotrophic factor (BDNF), which plays a crucial role in modulating synaptic plasticity for hippocampal-dependent cognitive functions, in 20 healthy male subjects (mean age 22.7 ± 2.3 years; mean BMI 24.9 ± 1.5 kg/m^2^) [12]. These findings suggest benefits of reduced fat intake on markers of AD; however, the research team did not investigate particular fatty acids such as the omega-3s.

A study on dietary polyunsaturated fats investigated the effect of a diet low in linoleic fatty acid on the plasma levels of oxidized linoleic acid metabolites (OXLAMs) [13]. Plasma OXLAMS are raised in AD and may relate to the disease progression [59]. Of interest, a separate study by this group showed a decrease in plasma OXLAMS in response to a 12-week intervention with a diet low in linoleic acid in chronic headache patients [14]. Further dietary interventions of longer duration should be conducted in at risk populations to clarify the effect of dietary fats, in particular omega-3 fatty acids, on risk of AD.

## 8. Role of Fatty Acids in Brain Function

### 8.1. Long Chain Omega-3 Fatty Acids and Brain Function

Dietary intake of DHA is required for normal neurodevelopment and brain health, particularly during prenatal brain development [60]. DHA is incorporated in large amounts into foetal brain through fatty acid transport protein-4 (FATP 4) [61]. DHA that is transferred from the maternal circulation to foetal brain plays a crucial role in brain growth especially with regard to synaptogenesis [62, 63]. However, the amount of maternal DHA in the synapses and neural membranes depends on the dietary intake [64–66]. The essential DHA is selectively enriched in neuronal tissues especially in neuronal and synaptic membranes, oligodendrocytes, and also subcellular particles such as myelin and nerve endings [67–69]. With aging, and especially among patients with AD, DHA levels in the brain tend to decrease [70].

### 8.2. Molecular and Cellular Mechanisms Underlying DHA Effects

DHA has been shown to have a crucial role in regulating neural gene expression [71]. Previous studies have shown that DHA acts as an endogenous ligand for retinoic acid receptors (RAR) and retinoid x receptors (RXR) [72]. RAR and RXR have been shown to decrease with age and these receptors are associated with age-related memory deficits. Dyall and colleagues [72] suggest that a reversal in the decrease of RAR and RXR following DHA supplementation could alleviate the memory deficits and increase neurogenesis. In patients diagnosed with AD, significantly lower DHA levels were detected in blood plasma and brain [73, 74]. This not only could be due to lower dietary intake of omega-3 fatty acids, but it also could be attributed to increased oxidation of PUFAs [75, 76].

While preclinical evidence suggests that a diet enriched with DHA reduces amyloid formation in dementia with AD [77, 78], clinical trials have yielded limited or negative results to date. Dietary supplementation of DHA has been shown to increase the levels of hippocampal BDNF [79]. Akbar and coworkers [70] provided additional evidence that DHA is highly enriched in neuronal membranes and that it facilitates the activation of protein kinase B (PKB), also known as Akt, via an increase in phosphatidylserine. Akt signalling is a critical pathway in neuronal survival. Activation of Akt, thus, can cause an increase in BDNF which further strengthens synaptic plasticity and cell survival. Furthermore, Calon and colleagues [80] found that a diet rich in DHA activates Ca^2+^/calmodulin-dependent protein kinase (CaMKII). This signalling cascade is critical for learning and memory and plays a crucial role in induction and maintenance of long-term potentiation in hippocampus [80, 81].

Studies also suggest that DHA modulates multiple cellular functions including enhanced membrane fluidity of amyloid precursor protein (APP) and a shift towards non-amyloidogenic processing, which inhibits *α* and *β* secretase, thereby reducing amyloid-*β* release [82]. Because of the influence of DHA on membrane fluidity, it has been speculated that DHA has significant impact on neural membrane function. DHA is suggested to facilitate N-methyl-D aspartate (NMDA) responses [83] and block K^+^ channels [84], which results in long-term potentiation, crucial for synaptic modification for long term memory and learning [83]. Omega-3 supplementation, DHA in particular, has also been shown to modulate gene expression at the transcription level, for example, by activating peroxisome proliferator-activated receptor (PPAR) family members [85] and the mRNA stability of several enzymes associated with glucose and lipid metabolism [86]. Studies in rodents indicate that treatment with fish oil resulted in overexpression of genes related to synaptic plasticity, signal transduction, energy metabolism, and regulatory proteins [87–89]. While interest in the underlying molecular and cellular mechanisms by which DHA exerts its beneficial effects in neurodegenerative conditions such as AD continues, the exact mechanisms are not clearly understood.

### 8.3. DHA Depletion and Cognitive Impairment

Studies in animal models of AD suggest that deficiency of DHA in neural tissue leads to behavioural deficits, ultimately leading to neurodegeneration and cognitive dysfunction similar to that in patients with AD [90–92]. Furthermore, experimental evidence suggests that DHA decreases with age, particularly in regions of the hippocampus which are crucial for higher brain functions such as memory formation and cognition [90, 93]. Decreased DHA levels are reported to detrimentally affect the major excitatory neurotransmitter, glutamate, which contributes to the integrity of brain function in learning memory performance [69].

## 9. Omega-3 Fatty Acid Supplementation Trials in Patients with Impaired Cognition and Alzheimer's Disease

In addition to health benefits of omega-3 supplementation in other settings, a number of studies report using omega-3 supplementation in (early) AD [94]. Epidemiological and preclinical studies indicate that consumption of long chain omega-3 PUFAs (omega-3 fatty acids) may slow cognitive decline and prevent the progression of mental health disorders such as AD. The relationship between mental health disorder and omega-3 fatty acids has been shown by lower levels of omega-3 fatty acids in the erythrocyte membrane or plasma of the patients suffering from neurodegenerative disorder as compared with healthy people [95–97]. Serum samples of patients with AD have also been reported with less than half the level of DHA compared to healthy individuals [98]. Importantly, however, very few randomized controlled trials have been conducted to ascertain the beneficial role of omega-3 fatty acids in prevention and progression of neurodegenerative diseases such as AD.

Controlled studies using omega-3 fatty acid supplementation in patients diagnosed with MCI (a precursor to early AD) or AD are few in number. To date, controlled studies conducted on patients with MCI and supplemented with omega-3 fatty acids (summarised in Table 3) suggest a positive effect on cognitive performance following supplementation ranging from 3 to 12 months. Kotani and colleagues [16] demonstrated that supplementation with 240 mg/day of DHA and 240 mg/day arachidonic acid significantly improved immediate memory and attention scores in adults with MCI, but not in 8 patients with AD who were given the same dose of supplementation for the same duration. These results are similar to the study conducted by Chiu and colleagues [15], who also reported improvements in MCI, but not AD, patients following omega-3 fatty acid supplementation over 24 weeks. It should be noted that one study reported no significant prevention of cognitive decline in older people with MCI over 6 months [19]. The researchers speculated that the lack of effect could be due to the low dose of DHA/EPA employed (180 mg DHA + 120 mg EPA of omega-3 PUFAs).

Most of the omega-3 supplementation studies in AD patients (summarised in Table 4) show no significant improvement in AD measures, except for Freund-Levi and colleagues [21], who conducted the largest trial to date (*n* = 174). These workers reported that supplementation with 1.72 g DHA + 600 mg EPA per day for 6 months did not show any improvement in cognitive decline in AD patients. However, in a very small subgroup of patients (*n* = 27) diagnosed with the mildest form of AD, a significant reduction in cognitive decline rate was observed compared to the placebo group. Another study conducted by Boston and coworkers [20] reported no difference in rate of cognitive decline between AD patients taking 1 g of ethyl-EPA daily for 3 months compared to placebo. A positive effect (*P* = 0.02) was seen in carer's visual analogue rating; however, the authors suggest that this result could be biased, as carer's were aware of the treatment regime. Nevertheless, the outcomes of this trial warrant further investigation in the form of a double-blinded study. Extending the duration of the supplementation period in AD patients has also been suggested.

In summary, results from controlled studies conducted over the last 10 years (summarised in Tables 3 and 4) suggest that nutritional intervention with omega-3 fatty acids is beneficial only in the earlier stages of cognitive impairment. Controlled studies on patients with well-established AD using both low and high doses of omega-3 fatty acids have not shown any improvement; this could be due to suboptimal levels of omega-3 fatty acids reaching the brain or the fact that the intervention is too late. The longest supplementation trial in AD patients reported to date is 18 months [23], although most have been for 6 months duration. Further studies of longer duration and with larger subject populations could provide more insight as to the therapeutic benefits of omega-3 fatty acids in slowing cognitive decline in AD patients. There is also good evidence from epidemiological studies that a Mediterranean-style dietary pattern (rich in plant derived ALA and LC-PUFA from fish and seafood) may be protective for neurodegenerative diseases such as AD and Parkinson's disease [99], and recent results from the Spanish PREDIMED study demonstrated improved cognition in high vascular risk participants who followed the Mediterranean diet compared with those on the low fat diet [100].

## 10. Conclusions and Future Directions

This review has highlighted the lack of consistent evidence for the potential of nutraceuticals and pharmacotherapies to delay the progression of AD. Evidence for a single nutrient therapy is inconsistent. Therefore, it appears that the overall quality and composition of the diet also contribute to protection against AD and dementia. The strongest evidence in support of nutrition preventing cognitive decline in AD is for long chain omega-3 fatty acids. Primarily, this is because long chain omega-3 has shown promising potential to ameliorate low grade inflammation in the early stages of this neurodegenerative disease.

Potential future directions for this field deserve attention in areas covering experimental design, dietary food guidelines, and targeting treatment for patients based on the stage of their disease. In particular, we highlight the need for the following: (1) Large, high quality randomised control trials with omega-3 fatty acid supplementation for a longer duration, possibly 18–24 months, to measure cognitive performance in Alzheimer's Disease Assessment Scale. Importantly, omega-3 doses need to achieve therapeutic concentration levels in the brain. These trials could offer long-term assessment of the effect of omega-3 on delaying the progression of cognitive decline associated with AD. (2) Standardized dietary guidelines for marine and plant-based omega-3 consumption are required and regular followup in patients with mild cognitive impairment is needed to ascertain if dietary intake could delay or reverse any deleterious effects that could progress to dementia or AD. (3) Finally, studies need to target people with mild cognitive impairment and early to moderate AD with low plasma levels of omega-3 at baseline.

## Figures and Tables

**Table 1:** Disease progression in Alzheimer's disease (AD).

Stage	Clinical trajectory
Early preclinical AD (changes begin 10–20 yrs before symptoms)	(i) Degeneration in hippocampus (where short-term memory is converted to long-term memory) (ii) Neuronal loss leads to shrinkage (iii) Memory loss is the first sign of AD

Mild-moderate AD	(i) Mild: memory loss, confusion, poor judgment, mood changes, anxiety (ii) Moderate: increased memory loss and confusion, problem recognizing people, difficulty with language, repetitive statements

Severe AD	(i) Extreme shrinkage of brain (ii) Patients completely dependent on others for care (iii) Weight loss, seizures, skin infections, moan/groaning, loss of bladder, and bowel control (iv) Death usually occurs from aspiration pneumonia or other infections

**Table 2:** Summary of observational and dietary intervention studies in patients with Alzheimer's disease.

Reference	Study design Duration Population (*n*, mean age/age range)	Dietary intake measures	Measures of cognitive decline	Outcome
[3]	Longitudinal study 18 mths “early stage” AD (36, ≥65 yrs) cognitively intact (58, >65 yrs)	Food records 24-hr diet recalls	MMSE score or Reisberg Global Deterioration Scale	Nutrient intakes significantly lower in patients with early stage AD included omega-3 fatty acids and omega-6 fatty acids

[4]	Longitudinal study 3.9 yrs cognitively intact (2148, ≥65 yrs)	FFQ	Neuropsychological battery and evidence of cognitive deficit	Dietary pattern exhibiting a lower risk on AD incidence included higher intake of nuts and fish

[5]	Longitudinal study 21 yrs healthy participants (1449, 65–79 yrs)	FFQ	DSMMD, 4th edition	Polyunsaturated fats associated with decreased rates of dementia and AD. Saturated fats associated with an increase in rates of dementia and AD

[6]	Case-cohort study (266, 65–100 yrs) 42 with dementia 30 with possible/ probable AD	FFQ	MMSE, Trail making test–part B, HVRT, CFT, BSRT	Reduction in odds of developing AD in those with the highest tertile of dietary DHA. Fish intake associated with lower odds of developing AD, but did not reach statistical significance

[7]	Prospective cohort 4 yrs nondemented (980, >65 yrs)	FFQ	DSMMD, 4th Edition	Consumption of up to three servings of wine daily was associated with a lower risk of AD in elderly

[8]	Longitudinal study 4 yrs nondemented (2258, 77 yrs)	FFQ	DSMMD, Revised 3rd Edition	Higher adherence to the MD was associated with lower risk for AD

[9]	Systematic review 6 studies various (16995, various)	FFQ	Various	Higher adherence to the MD associated with a lower risk of dementia or AD than subjects in the lowest tertile of adherence

[10]	Meta-analysis 3 studies various (133626, various)	Various	Various	Adherence to MD led to a 13% lower incidence of neurodegenerative diseases such as AD

[11]	Dietary intervention 4 wks healthy participants (20, 69 yrs) aMCI (29, 68 yrs)	Daily food diary	Immediate and delayed memory test, executive function test, motor speed test	Diet low in saturated fat and GI decreased markers associated with risk of AD compared to the high fat and GI intervention

[12]	Dietary intervention single meal healthy males (20, 23 yrs)	Single meal delivered	Not measured	High fat meal caused a decrease in circulating brain-derived neurotrophic factor (BDNF)

[13, 14]	Dietary intervention 12 wks headache patients (56, 41 yrs)	24-hr diet recalls	Not measured	Lowering dietary linoleic fatty acids significantly reduced levels of plasma oxidated linoleic acid metabolites (OXLAMs)

aMCI = amnestic mild cognitive impairment; BSRT = Buschke-Fuld Selective Reminding Test; CFT = Category Fluency Test; DSMMD = Diagnostic and Statistical Manual of Mental Disorders; FFQ = Food Frequency Questionnaire; HVRT = Heaton Visual Reproduction Test; MD = Mediterranean Diet; MMSE = Minimental State Examination.

**Table 3:** Summary of DHA/EPA dietary intervention trials in patients with mild cognitive impairment (MCI) in the last 10 years.

Reference	Clinical trials with MCI patients (*n*, mean age)	Dosage of DHA/EPA per day	Trial duration and design	Measures	Outcome
[15]	Patients with MCI (23, 74 yrs)	0.72 g DHA + 1.08 g EPA or placebo	6 mths randomized double-blinded placebo-controlled trials	ADAS-cog.; CIBIC plus	Significant improvement in ADAS-cog; in patients with MCI after omega-3 supplementation

[16]	Patients with MCI (23, 68 yrs)	240 mg DHA + 240 mg AA or placebo	3 mths, placebo controlled trial	Japanese version of RBANS (5 cognitive domains)	Improvement of immediate memory and attention in omega-3 supplemented group

[17]	Elderly persons with MCI (36, 66 yrs)	1.3 g DHA + 0.45 mg of EPA or placebo	12 mths, randomized double-blinded placebo controlled trial	RAVLT, MMSE, CDT, WAIS-R	Significant improvement in cognitive function in omega-3 supplemented group

[18]	Elderly patients suffering from MCI (11, 85 yrs)	1.4 g DHA + 572 g EPA or placebo	3 mths, randomized double-blinded placebo controlled trial	MMSE	Significant improvement in MMSE, semantic verbal fluency, and olfactory sensitivity assessment in omega-3 supplemented group

[19]	Older people with MCI (100, 74 yrs)	180 mg DHA + 120 mg EPA or placebo	6 mths, randomized double-blinded placebo controlled trial	MMSE, AMT	Low prescription dose had no effect on cognitive function in omega-3 supplemented group

AA = arachidonic acid; DHA = docosahexaenoic acid; EPA = eicosapentaenoic acid; MMSE = Minimental State Examination; ADAS-cog. = Cognitive Portion of the Alzheimer's Disease Assessment Scale; CIBIC plus = Clinician's Interview-Based Impression of Change Scale; RBANS = Repeatable Battery for the Assessment of Neuropsychological Status; RAVLT = Rey's Auditory Verbal Learning Test; CDT = Clock Drawing Test; WAIS-R = Wechsler Adult Intelligence Scale; AMT = Abbreviated Mental Test.

**Table 4:** Summary of DHA/EPA dietary intervention trials in patients with Alzheimer's disease (AD) in the last 10 years.

Reference	Clinical trials with AD patients (*n*, mean age)	Dosage of DHA/EPA per day	Trial duration and design	Measures	Outcome
[20]	Mild to moderate AD (22, 81 yrs)	1 g ethyl-EPA or placebo	6 mths; 12 wks without treatment, followed by 12 wks with treatment	MMSE, ADAS-cog.	NS difference between treatment and baseline, small improvement in carer's analogue rating (*P* = 0.02)

[16]	Patients with AD (8, 67 yrs)	240 mg DHA + 240 mg AA or placebo	3 mths parallel design	Japanese version of RBANS (5 cognitive domains)	NS improvement seen postsupplementation

[21]	Mild AD patients (178, 74 yrs)	1.72 g DHA + 600 mg EPA or placebo	6 mths parallel design	MMSE, ADAS-cog.	Positive effects of omega-3 supplementation seen only on patients with very mild AD

[15]	Mild to moderate AD (23, 74 yrs)	0.72 g DHA + 1.08 g EPA or placebo	6 mths, randomized double blinded placebo controlled trial	ADAS-cog.; CIBIC-plus	NS difference seen between placebo and omega-3 supplemented group

[22]	AD patients on acetylcholine esterase treatment (204, 74 yrs)	1.72 g DHA + 600 mg EPA or placebo	6 mths parallel + 6 mths cross-over to fish oil	DAD, CGB, MADRAS, NPI	NS effect between omega-3 supplemented placebo group on neuropsychiatric symptoms, positive effect on depressive symptoms

[23]	Mild to moderate AD (295, 76 yrs)	2 g DHA or placebo	18 months, randomized double blinded placebo controlled trial	ADAS-cog, CDR, MMSE; (brain MRI in sub pop. *n* = 102)	NS difference in rate of cognitive and functional decline, no effect on total brain volume

AA = arachidonic acid; DHA = docosahexaenoic acid; EPA = eicosapentaenoic acid; DAD = Disability Assessment for Dementia Scale; CGB = Caregiver Burden Scale; MADRS = Montgomery-Asberg Depression Rating Scale; NPI = Neuropsychiatric Inventory; MMSE = Minimental State Examination; ADAS-cog. = Cognitive Portion of the Alzheimer's Disease Assessment Scale; CIBIC plus = Clinician's Interview-Based Impression of Change Scale; RBANS = Repeatable Battery for the Assessment of Neuropsychological Status; NS = Nonsignificant; MRI = Magnetic Resonance Imaging; CDR = Clinical Dementia Rating.

## Abbreviations

Amyloid-*β*: Amyloid Beta (protein)
ALA: Alpha-Linolenic acid
AD: Alzheimer's disease
DHA: Docosahexaenoic acid
EPA: Eicosapentaenoic acid
MCI: Mild cognitive impairment
PUFAs: Polyunsaturated fatty acids.
